# Transmission and mortality risk assessment of severe fever with thrombocytopenia syndrome in China: results from 11-years' study

**DOI:** 10.1186/s40249-022-01017-4

**Published:** 2022-09-04

**Authors:** Qiulan Chen, Dong Yang, Yanping Zhang, Mantong Zhu, Ning Chen, Zainawudong Yushan

**Affiliations:** 1 Key Laboratory of Surveillance and Early-Warning on Infectious Diseases, Chinese Center for Disease Control and Prevention, Beijing, China; 2 Changsha Center for Disease Control and Prevention, Changsha, China; 3 School of Public Health, Guangxi Medical University, Nanning, China; 4 Chinese Center for Disease Control and Prevention, Changbai Road, Changping, Beijing, 102206 China

**Keywords:** Severe fever with thrombocytopenia syndrome, Cluster, Human-to-human transmission, Transmission risk, Secondary attack rate, Blood contact, Relative risk, Epidemiological characteristics, Mortality, China

## Abstract

**Background:**

The transmission and fatal risk of severe fever with thrombocytopenia syndrome (SFTS), an emerging infectious disease first discovered in China in 2009, still needed further quantification. This research aimed to analyze the SFTS clusters and assess the transmission and mortality risk for SFTS.

**Methods:**

Both epidemiological investigation and case reports regarding SFTS clusters in China during 2011–2021 were obtained from the Public Health Emergency Information Management System of the Chinese Center for Disease Control and Prevention Information System. The transmission risk was evaluated by using the secondary attack rate (SAR) and relative risk (*RR*). Mortality risk factors were analyzed using a logistic regression model.

**Results:**

There were 35 SFTS clusters during 2011–2021 involving 118 patients with a fatality rate of 22.0%. The number of clusters annually increased seasonally from April to September. The clusters mainly occurred in Anhui (16 clusters) and Shandong provinces (8 clusters). The SAR through contact with blood or bloody fluids was much higher than that through contact with non-bloody fluids (50.6% vs 3.0%; *χ*^*2*^ = 210.97, *P* < 0.05), with an *RR* of 16.61 [95% confidence interval (*CI*): 10.23–26.97]. There was a statistically significant difference in the SAR between exposure to the blood of a deceased person during burial preparation and exposure to the living patients’ blood (66.7% vs 34.5%; *χ*^*2*^ = 6.40, *P* < 0.05), with an *RR* of 1.93 (95% *CI*: 1.11–3.37). The mortality risk factors were a long interval from onset to diagnosis [odds ratio (*OR*) = 1.385), 95% *CI*: 1.083–1.772, *P* = 0.009) and advanced age (*OR*: 1.095, 95% *CI*: 1.031–1.163, *P* = 0.01).

**Conclusions:**

The SFTS clusters showed a high mortality rate and resulted in a high SAR. Contact with a bleeding corpse was associated with a higher infection risk, compared with contacting the blood from living patients. It is important to promote early detection and appropriate case management of patients with SFTS, as well as improved handling of their corpses, to prevent further transmission and mortality.

**Graphical abstract:**

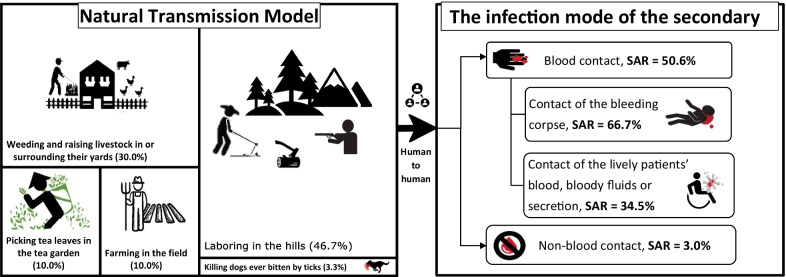

## Background

In 2006, severe fever with thrombocytopenia syndrome (SFTS), which is characterized by fever and thrombocytopenia, was discovered and successively reported in rural areas in central and eastern China, including Henan, Hubei, Anhui, and Jiangsu provinces. It is also characterized by obvious bleeding tendencies accompanied by leukopenia and multiple organ dysfunction [1]. In 2009, Chinese researchers isolated the virus from patients in Henan and Hubei and termed it as SFTS virus (SFTSV) [2], which was subsequently renamed as *Dabie bandavirus*. Tick bites are the main transmission route for SFTSV, followed by contact with the blood and bloody secretions of the patients [3, 4]. Subsequently, Japan, the Republic of Korea, and Vietnam have reported patients with SFTS [5–7]. Worldwide, the case fatality rate (CFR) of SFTS ranges from 15.1 to 50% depending on delayed hospital admission, high viral load, age, and patient comorbidities/complications [8]. The incubation period of SFTS through human-to-human transmission is 3–15 days, with a median of 10 days [9]. However, the pathogenesis of SFTS remains unclear; moreover, no specific drugs or effective vaccines are available. In 2017, the World Health Organization listed SFTS as one of the world's top emerging infectious diseases that could cause a pandemic or that currently lacked medical resolution [10].

In China, SFTSV usually causes sporadic cases in rural areas; however, it can occasionally develop clusters, which poses a great threat to public health by causing death and infecting secondary patients. Previous studies had demonstrated the high CFR and risk of contacting the bleeding corpse during final preparations for a single cluster [2, 4, 9–16]. Only a few studies have quantitatively assessed the human-to-human transmission risk among SFTS clusters [17]. However, the risk factors for fatal outcomes among SFTS clusters based on a multivariate model from a public health perspective, as well as comparison of the transmission risk between the routes of contacting the bleeding corpse and blood from living patients, remain unclear. This could be attributed to data unavailability. There is insufficient awareness regarding SFTS and the need to decontaminate the corpses of patients with SFTS in rural China. Accordingly, we aimed to explore the mortality risk factors among SFTS clusters, as well as to quantify the risk of different transmission routes (blood contact vs non-blood contact; contact with a bleeding corpse vs contact with the blood from living patients).

## Methods

### Key terminology

Based on the national guideline for the prevention and control of SFTS [18], which was issued in 2010 by the Chinese Ministry of Health, patients with confirmed SFTS were defined as patients who worked, lived, or traveled through hillsides, forest areas, mountains, or other places during the epidemic season; or those with a history of being bitten by a tick within 2 weeks of disease onset with clinical manifestations such as fever, decreased peripheral blood platelet and leukocyte counts, and at least one of the following laboratory findings: (1) detection of SFTSV RNA; (2) seroconversion or > fourfold increase in the specific antibody to SFTSV between the acute and convalescent serum samples; or (3) isolation of SFTSV from the case specimens.

SFTS clusters [18] refer to Public Health Emergency Events in which two or more cases occurred among people living, working, or traveling in the same village or throughout the same hillside, forest, tea garden, scenic spot, or where at least one case occurred among close contacts of the index case.

The index case [16] was defined as the first case identified at the onset of an epidemiological investigation, where the person was infected with SFTSV through exposure to ticks or other routes.

The secondary attack rate (SAR) refers to the percentage of cases among the total number of susceptible contacts occurring between the shortest and longest incubation periods of certain infectious diseases after exposure to a primary case. It is calculated as follows:

SAR (%) = number of patients among susceptible contacts between the shortest and longest incubation periods/total number of susceptible contacts × 100%.

### Data source and data collection

Based on the national guideline for prevention and control of SFTS [18], SFTS is described with reference to a category B “notifiable infectious disease” in the mainland of China given that it was first identified in 2009. All healthcare facilities are required to report both patients with suspected and confirmed SFTS within 24 h of detection to the National Notifiable Infectious Diseases Surveillance System (NNIDSS), which is a subsystem of the China Disease Control and Prevention Information System (CDCPIS) that tracks patient information (e.g., clinical categorization). In addition, the local Centers for Disease Control and Prevention are required to report SFTS clusters to the National Public Health Emergency Event Surveillance System (PHEESS), which is another subsystem of CDCPIS that focuses on cluster investigation.

The internet-based PHEESS comprises two modules: (1) a structured database with data items including, but not limited to, time, location, cluster settings (e.g., tea garden, hospital), infection route, numbers of exposed (including close contacts identified through cluster investigation), infected individuals, and deaths; moreover, (2) additional information that does not fit into any specific database category is included in the unstructured narratives attached to the PHEESS reports. Such information includes epidemic curves (by symptom onset, as photos), tables (listing the patients’ demographic characteristics), laboratory test results (IgG titer and whether the virus was isolated), and control measures (hospital infection control measures and environmental disinfection). The completeness and quality of these narratives varied across municipalities.

A retrospective study was conducted on SFTS clusters reported to the PHEESS between January 1, 2011, and December 31, 2021. Here, both structured data and nonstructured narratives of all SFTS clusters reported during this period were downloaded from the PHEESS and analyzed. Clusters (*n* = 17) that resulted in secondary patients via human-to-human transmission routes were included when calculating the SARs of different infection modes. All the data were permitted to use by Chinese Center for Disease Control and Prevention, and none of the data in relation to personal identify were disclosed.

### Data management and analysis

Information provided in the unstructured narratives was abstracted for temporal, spatial, and demographic parameter indicators before being summarized and analyzed. Descriptive epidemiological methods were used to describe the temporal and spatial distribution of clusters and the demographic characteristics of involved patients. The transmissibility and relative risk (*RR*) of different infection routes were evaluated based on the SARs, including all 17 clusters with human-to-human transmission. We explored risk factors by analyzing differences in age, sex, the time interval from onset to confirmation, occupation, and infection routes between deceased and cured patient groups. The normality test was used for between-group comparisons of age and the time interval from onset to confirmation. The *t*-test and Wilcoxon rank-sum test were used for between-group comparisons in case of normal and non-normal distributions, respectively. The chi-square test was used for between-group comparisons of age, occupation, and contact routes. A multivariate logistic regression model was used to explore mortality risk factors in the SFTS clusters. Significant variables in the univariate analysis were included in the multivariate model as independent variables. All statistical analyses were performed using R software (version 4.1.3; R Foundation for Statistical Computing, Vienna, Austria) and Microsoft Excel (version 2019; Microsoft Corporation, Redmond, WA, USA).

## Results

### Temporal and spatial distribution of SFTS clusters in China

Between 2011 and 2021, 35 SFTS clusters were reported in China, which involved 118 patients, of which 26 died (CFR = 22.0%). The CFR was higher among female patients (31.4%, 16/51) than among male patients (14.9%, 10/67). Moreover, the CFR was higher among patients aged ≥ 60 years (35.3%, 24/68) than among patients aged < 60 years (4.0%, 2/50).

**Fig. 1:**
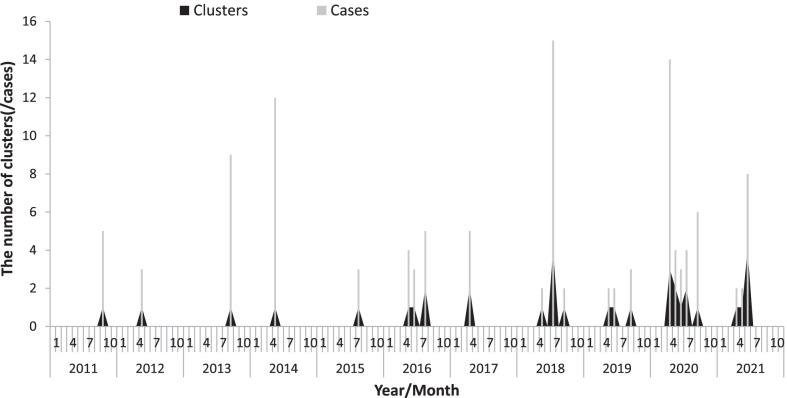
The seasonality of SFTS clusters in China from 2011 to 2021. SFTS, severe fever with thrombocytopenia syndrome

There was an annual increase in the incidence of SFTS clusters, which was the highest in 2020 (*n* = 9), followed by 2018 and 2021 (*n* = 6). The incidence rates of clusters in April, May, June, July, August, and September were 17.4%, 22.9%, 20.0%, 17.1%, 8.6%, and 11.4%, respectively (Fig. 1), which indicated an epidemic seasonality during summer and autumn.

The SFTS clusters were reported in the provinces of Anhui (*n* = 16), Shandong (*n* = 8), Jiangsu (*n* = 4), Zhejiang (*n* = 3), Hubei (*n* = 2), and Hunan (*n* = 2). The number of individuals involved in each cluster ranged from two to twelve persons, with the median number being two. The sex ratio (male/female) of the included patients was 1.31∶1 (67/51). The age range and mean age of the patients were 18–84 years and 59.0 ± 14.2 years, respectively.

### Infection routes and venue of SFTS clusters in China

The infection routes of the index patients in 14 and 16 clusters were tick bites and suspected tick bites, respectively, with those of the remaining five clusters being unknown. The index patients were exposed to the ticks by picking tea leaves in the tea garden (10.0%, 3/30); farming in the field (10.0%,3/30); weeding and raising livestock in yards or their surroundings (30.0%, 9/30); laboring in the hills (27.0%, 8/30), including hunting, cutting wood, digging trees, picking fruits, and looking for medical herbs; and contact with the blood of a dog infected by tick bites (3.3%, 1/30) or both laboring in the hills and weeding and raising livestock in yards or their surroundings (20.0%, 6/30).

**Fig. 2:**
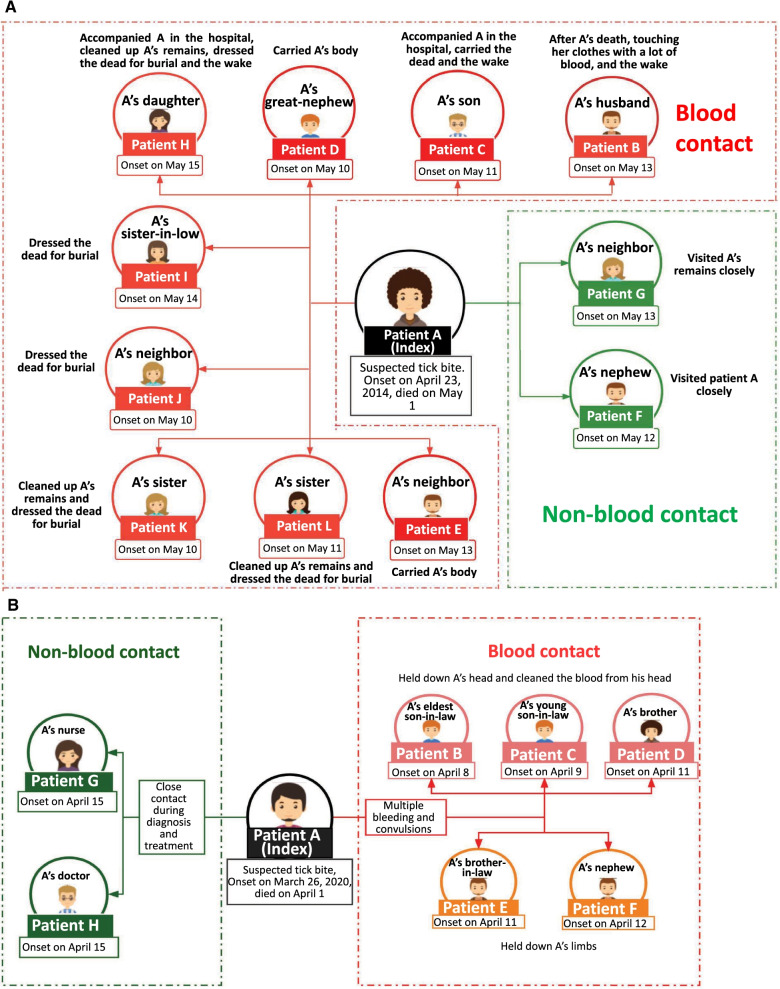
**A** Transmission routes for one SFTS cluster in Anji County, Zhejiang Province, 2014. **B** Transmission routes for one SFTS cluster in Hanshan County, Anhui Province, 2020. **A** Patient A was the index patient and died of massive bleeding while being transferred from hospital to home. The patient had infected 11 secondary patients (Patient B–Patient L); among them, nine patients were infected by blood contact while the other two patients were infected through inhalation of *Brucella*-containing aerosol in a confined mourning room, without direct contact with the patient or other possible exposure. All the secondary patients did not wear personal protection equipment during the exposure. The index patient had been exposed to a tick bite while picking tea leaves on the tea garden. The serum positive detection rates of SFTSV IgG were 1.6% and 2.0% in healthy people and ducks, respectively, living in the village where the index patient lived. **B** The index patient (**A**) was a 51-year-old male farmer who was infected through contact with the blood of a dead dog that had been bitten by ticks. He had infected seven secondary cases. Specifically, five family members and relatives were infected through blood contact while a nurse and a doctor were infected through non-blood contact. *SFTS* severe fever with thrombocytopenia syndrome, *SFTSV* severe fever with thrombocytopenia syndrome virus

There were 17 clusters that resulted in secondary patients through the index patients via human-to-human transmission. Among them, four occurred in hospitals, three occurred in homes, and the other ten occurred in both hospitals and patients’ homes. The secondary patients included the primary cases’ family members, relatives, doctors and nurses, and even fellow villagers. The exposure routes comprised blood contact (i.e. contact with blood or bloody fluids and secretions from the patients) and non-blood contact (i.e. contact with patients’ fluids or secretions other than blood or inhalation of *Brucella*-containing aerosol) while providing care for the index patients, transferring dying patients with hemorrhagic clinical manifestation, or during burial preparations. Nosocomial infection occurred in two clusters, which involved one doctor and one nurse in each cluster. The doctor was exposed while performing a sputum suction operation without a closed sputum suction tube and/or touching the patient’s blood without personal equipment protection (PEP). The nurse was infected while changing sheets contaminated with fresh blood from the same patient; however, she wore gloves without wearing mask, indicating possible infection by aerosol inhalation. Another doctor and nurse were infected through non-blood contact while providing medical care without any PEP to another patient. The transmission routes of two clusters that involved eleven and seven secondary patients with nosocomial infection are illustrated in Fig. 2A and B, respectively.

Among the remaining 18 clusters that caused no human-to-human transmission, eleven, six, and one occurred in the village living environment, fields, and tea garden, respectively. Further details are provided in Table 1.

**Table 1:** Characteristics of SFTS clusters in China, 2011–2021

Serial code	Time	Location	Cluster scale, *n*	Death cases, *n*	Infection route of index case	Human to human transmission	Secondary cases, *n*	Place
1	October 2011	Rongcheng city, Shandong province	5	1	Tick bite	Yes	4	Hospital
2	May 2012	Wuhan city, Hubei province	3	2	Tick bite	Yes	2	Home
3	September 2013	Penglai city, Shandong province	9	2	Tick bite	Yes	8	Hospital and home
4	May 2014	Huzhou city, Zhejiang province	12	1	Suspected tick bite	Yes	11	Hospital and home
5	October 2015	Chuzhou city, Anhui province	3	1	Not known	Yes	2	Hospital and home
6	May 2016	Yantai city, Shandong province	4	1	Suspected tick bite	No	–	Living environment
7	July 2016	Suzhou city, Jiangsu province	3	1	Suspected tick bite	Yes	2	Hospital and home
8	August 2016	Maanshan city, Anhui province	3	1	Not known	No	–	Living environment
9	August 2016	Tongling city, Anhui province	2	1	Tick bite	Yes	1	Home
10	April 2017	Tongling city, Anhui province	2	0	Not known	No	–	Field
11	April 2017	Suizhou city, Hubei province	3	1	Tick bite	Yes	2	Hospital and home
12	May 2018	Maanshan city, Anhui province	2	0	Suspected tick bite	No	–	Field
13	July 2018	Shaoxing city, Zhejiang province	4	2	Suspected tick bite	Yes	3	Hospital
14	July 2018	Nanjing city, Jiangsu province	7	2	Suspected tick bite	Yes	6	Hospital and home
15	July 2018	Maanshan city, Anhui province	2	0	Suspected tick bite	No	–	Living environment
16	July 2018	Weihai city, Shandong province	2	1	Not known	No	–	Living environment
17	September 2018	Weihai city, Shandong province	2	1	Suspected tick bite	Yes	1	Hospital
18	May 2019	Chuzhou city, Anhui province	2	0	Tick bite	No	–	Field
19	June 2019	Zhangjiajie city, Hunan province	2	0	Tick bite	No	–	Living environment
20	September 2019	Lianyungang city, Jiangsu province	3	2	Tick bite	Yes	2	Hospital and home
21	April 2020	Tongling city, Anhui province	2	1	Suspected tick bite	No	–	Living environment
22	April 2020	Maanshan city, Anhui province	8	1	Tick bite	Yes	7	Hospital
23	April 2020	Anqing city, Anhui province	4	0	Tick bite	No	–	Tea garden
24	May 2020	Nanjing city, Jiangsu province	2	0	Suspected tick bite	No	–	Living environment
25	May 2020	Maanshan city, Anhui province	2	1	Suspected tick bite	Yes	1	Hospital
26	June 2020	Hefei city, Anhui province	3	0	Suspected tick bite	No	–	Living environment
27	July 2020	Jinhua city, Zhejiang province	2	0	Suspected tick bite	No	–	Field
28	July 2020	Chaohu city, Anhui province	2	0	Suspected tick bite	No	–	Living environment
29	September 2020	Huaihua city, Hunan province	6	1	Tick bite	Yes	5	Hospital and home
30	June 2021	Chaohu city, Anhui province	2	0	Tick bite	No	–	Living environment
31	June 2021	Maanshan city, Anhui province	2	0	Tick bite	No	–	Field
32	May 2021	Maanshan city, Anhui province	2	1	Tick bite	Yes	1	Hospital
33	April 2021	Changzhou city, Jiangsu province	2	0	Suspected tick bite	Yes	1	Home
34	June 2021	Weihai city, Shandong province	2	1	Suspected tick bite	No	–	Field
35	June 2021	Weihai city, Shandong province	2	0	Not known	No	–	Living environment

**Fig. 3:**
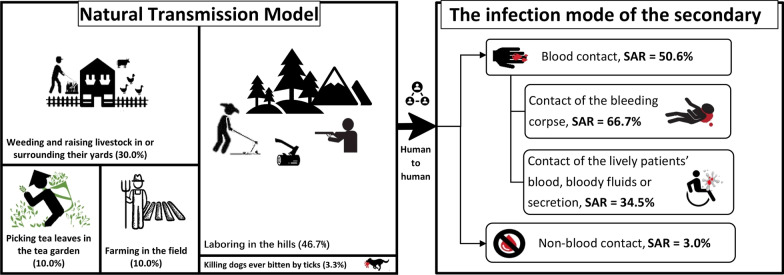
Transmission model and risk of different human-to-human transmission modes among SFTS in China. Note: The left picture describes the 30 index patients’ exposure ways to SFTSV. All were exposed during their routine laboring related with agriculture. There are six index patients exposed to confirmed or suspected tick bites during both laboring in the hills and weeding and raising livestock in yards or their surroundings. *SFTS* severe fever with thrombocytopenia syndrome, *SFTSV* Severe fever with thrombocytopenia syndrome virus, *SAR* the secondary attack rate

The median numbers of infected individuals among the clusters with and without secondary human-to-human transmission were 2.0 (2.0–2.0) and 3.0 (2.0–6.0), respectively (*U* = 71.00, *P* = 0.003). The transmission model of SFTS clusters with and without secondary human-to-human transmission are summarized in Fig. 3.

### Risk evaluation of different transmission modes among clusters that caused human-to-human transmission

Infection through blood contact showed a higher SAR than infection through non-blood contact [50.6% vs 3.0%, *RR* = 16.61, 95% confidence interval (*CI*): 10.23–26.67, *P* < 0.05]. Infection through contact with a bleeding corpse showed a higher SAR than infection through blood contact during hospital care (i.e., contact with a living patient’s blood, bodily fluids, or secretions) (66.7% vs 34.5%, *RR* = 1.93, 95% *CI*: 1.11–3.37, *P* < 0.05), as shown in Table 2 and Fig. 3.

**Table 2:** Relative risk between different transmission routes among SFTS clusters

Transmission route	Exposed population (*n*)	Secondary patients (*n*)	SAR (%)	*RR* (95% *CI*)	*χ*^2^	*P*
Blood contact	77	39	50.6	16.61 (10.23–26.97)	210.97	< 0.05
Non-blood contact	656	20	3.0	–		
Subtotal	733	59	8.0			
Contact of the bleeding corpse	33	22	66.7	1.93 (1.11–3.37)	6.40	< 0.05
Contact of the living patients’ blood, bloody fluids or secretion	29	10	34.5	–		
Subtotal	62	32	51.6			

*SAR* secondary attack rate, *RR* relative risk, – not applicable

### Mortality risk factors among clusters

Univariate analysis of risk factors revealed that longer time interval between onset and diagnosis (*U* = 796; *P* < 0.05), higher sex ratio (male/female) (*χ*^2^ = 4.56; *P* < 0.05), and older age (*t* = 6.09, *P* < 0.05) were observed in the group with dead patients than in that with cured patients. There was a significant between-group difference in the infection routes (*χ*^2^ = 11.51, *P* < 0.05) but not in occupation (*χ*^2^ = 0.04, *P* > 0.05). Further details are provided in Table 3.

**Table 3:** Univariate analysis of risk factors for death in SFTS clusters^*^

Study variables	*n* = 118	Death *n* = 26	Cured patients *n* = 92	*χ*^2^	*t*	*U*	*P*
Gender				4.56			< 0.05
Male	67 (56.8)	10 (38.5)	57 (62.0)				
Female	51 (43.2)	16 (61.5)	35 (38.0)				
Age^a^					6.09		< 0.05
Range	18–84	51–84	18–84				
Mean (SD)	59.1 (14.2)	69.2 (7.6)	56.3 (14.4)				
Occupation				0.04			> 0.05
Farmers	110 (93.2)	24 (92.3)	86 (93.5)				
Other occupations	8 (6.8)	2 (7.7)	6 (6.5)				
Transmission route				11.51			< 0.05
Tick-bite /suspected tick bite	51 (43.2)	18 (69.2)	33 (35.9)				
Blood contact^b^	39 (33.1)	3 (11.5)	36 (39.1)				
Non-blood contact^c^	18 (15.3)	2 (7.7)	16 (17.4)				
Not known	10 (8.5)	3 (11.5)	7 (7.6)				
Period from onset to diagnosis (days)^a^						796	< 0.05
Median (IQR)	3.0 (1.3–4.0)	3.5 (3.0–5.0)	2.0 (1.0–4.0)				

^a^Data are *n* (%) of case, unless otherwise indicated. Percentages may not total 100 because of rounding. SD, standard deviation. IQR, inter quartile range

^b^Blood contact refers to contacting the patients’ blood, bloody fluids or secretions and the bleeding corpse

^c^Non-blood contact refers to contacting the patients’ fluids or secretions other than blood

Statistically significant variables in the univariate analysis were included in the binary logistic regression model as independent variables. This model showed that the time interval from onset to diagnosis [odds ratio (*OR*) = 1.385; 95% *CI*: 1.083–1.722, *P* = 0.009] and old age (*OR* = 1.095; 95% *CI*: 1.031–1.163, *P* = 0.003) were mortality risk factors in these clusters. Specifically, the interval from onset to diagnosis and age were positively correlated with the mortality risk (Table 4).

**Table 4:** Logistic regression analysis of risk factors for death in SFTS clusters

Impacting factor	*β*	*S.E.*	Wald *χ*^*2*^	*P*	*OR*	95% *CI*
Period from onset to diagnosis (days)	0.326	0.125	6.754	0.009	1.385	1.083–1.772
Gender						
Male	1.00					
Female	0.533	0.544	0.961	0.327	1.705	0.587–4.953
Age	0.091	0.031	8.700	0.003	1.095	1.031–1.163
Transmission route						
Non-blood contact^a^	1.00					
Tick-bite/suspected tick bite	0.970	0.982	0.976	0.323	2.637	0.385–18.059
Blood contact^b^	− 0.015	1.136	0.000	0.990	0.985	0.106–9.127
Not know	− 0.148	1.273	0.013	0.908	0.863	0.071–10.447

*OR* odds ration, *CI* confidence interval

^a^Non-blood contact refers to contacting the patients’ fluids or secretions other than blood

^b^Blood contact refers to contacting the patients’ blood, bloody fluids or secretions and the bleeding corpse

## Discussion

This retrospective review of SFTS clusters reported in China from 2011 to 2021 found that they mainly occurred in Henan, Hubei, Anhui, and Shandong provinces. Moreover, the SFTS clusters showed significant seasonality, with peaks being observed during summer and autumn. The infection routes of the index and secondary cases were mainly tick bites and human-to-human transmission, respectively. Blood contact showed a higher transmission risk than that with non-blood contact, which is consistent with previous reports [4, 16]. Additionally, contact with a bleeding corpse showed a higher transmission risk than contact with a living patient’s blood. SFTS clusters caused rather high CFRs. In addition, advanced age and a long interval from onset to diagnosis were identified as mortality risk factors.

Ticks are the main transmission vectors of SFTS [19, 20]. The observed seasonality of SFTS clusters could be attributed to seasonal fluctuations in tick densities and human activities. Surveillance of biological vectors based on multiple sites has shown that the dominant tick species is *Haemaphysalis longicornis*; moreover, its activity shows obvious seasonality, beginning in spring and continuing through autumn [21, 22]. Ticks mainly inhabit mountainous hills or forest farms with rich vegetation; further, their growth and reproduction are affected by climatic factors, including temperature, humidity, and sunlight. Seasonal changes in these factors cause natural fluctuations in tick density. Outdoor activities, including farming, mowing, hunting, tea leaf picking, grazing, and traveling, mostly occur during summer and autumn. The high incidence of SFTS clusters in some cities in Shandong, Anhui, and Hubei provinces could be attributed to their mountainous and hilly topography, which provides ideal conditions for the growth and reproduction of ticks. Farmers living in mountainous and hilly areas have an increased chance of being exposed to tick bites since they often engage in agricultural labor, including farming, mowing, hunting, picking tea leaves, and herding; moreover, ticks living in the aforementioned endemic areas have a high SFTS infection rate [23]. SFTS clusters share the same ecological environmental characteristic of hilly landscapes; additionally, its key environmental risk factors include slope and maximum temperature of the warmest month; elevation; high coverages of woods, crops, and shrubs; and the vicinity of habitats of migratory birds [24, 25].

In our study, the reported SFTS clusters showed a substantially high CFR of 22.0%. However, the average annual CFR of SFTS cases nationwide in China during the same period was 5.1%; further, it considerably varied from 1.3% to 11.3% across the top seven endemic provinces in China based on the NNIDSS [26]. This discrepancy could be attributed to two main reasons. First, nationwide, SFTS usually presents as sporadic cases. Compared with sporadic cases, index patients among the clusters may have excreted higher viral loads, which resulted in higher CFRs. Second, due to the constraints of economic conditions and local culture, some critically ill patients were voluntarily discharged from the hospital and chose to die at home; therefore, they were not accounted for while determining the CFR if the local health system lacked follow-up mechanisms for outcome evaluation [27, 28]. For example, a large-scale single-center prospective study on 2096 SFTS reported a higher CRF (16.2%) than that reported by the national surveillance system [27].

Advanced age seems be a risk factor for SFTS mortality, which could be attributed to the fact that many older adults have underlying chronic diseases, decreased immunity, and an increased risk of severe infections [29]. Another risk factor for SFTS mortality was a long-time interval from onset to diagnosis, which may be related to the mechanism of SFTS pathogenesis [28, 29]. Early diagnosis and prompt treatment are crucial for reducing SFTS mortality. Other recommended interventions include active mass public health education in SFTS-endemic areas, improved diagnostic capacity of local medical and health institutions, and establishment of an effective referral system for patients with severe SFTS.

Contact with a bleeding corpse showed a higher transmission risk than contact with the blood of living patients. This may be attributed to the higher viral load of SFTSV excreted by critically ill dying patients than that by living patients. Our findings could provide further insight into the mechanisms underlying the transmission of SFTS as well as inform prevention and control strategies for SFTS in rural China. To our knowledge, this is the first study to compare the risk between exposure to bleeding corpses and exposure to blood and bloody fluids from living patients. Our findings demonstrate the importance of proper disposal of the corpses of patients who die from SFTS. According to local customs in rural China, family members, relatives, or villagers usually clean the body of the deceased and then dress it for burial, which inevitably leads to contact with the bleeding corpse. As aforementioned, in SFTS-endemic areas in rural China, especially remote and undeveloped areas, the family often prefer to take the critically ill patient home due to economic constraints and cultural customs [27, 28]. Patients with severe SFTS usually present with bleeding, including hemoptysis, hematemesis, gingival bleeding, nasal bleeding, hematochezia, and vaginal bleeding [27]. Accordingly, without effective personal protection equipment (PPE), family members or relatives can be easily infected through contact with blood and secretions while caring for the patients [30]. Similarly, this can result in community transmission through contact with a bleeding corpse while preparing the burial [4]. Endemic communities should be educated on how to utilize the necessary PPE to avoid direct contact with blood, bodily fluids, bloody secretions, and bleeding corpse. Additionally, patients’ caregivers should receive PPE training upon admission or confirmation of infection. Generally, there is a need to establish protocols for SFTS case management and corpse decontamination for patients who died of SFTS to avoid further transmission and mortality.

In addition, our findings demonstrated that SFTS causes nosocomial infections among medical staff. Therefore, medical staff should consistently wear PPE and adopt standard protocols when caring for patients with suspected or confirmed SFTS.

This study had several limitations. First, the data were obtained from China's PHESS, which may not reflect the real-world situation due to the sensitivity of the monitoring system and local reporting awareness. Second, we did not analyze the risk factors of the index patients due to incomplete data information in different regions. However, the database used in this study is currently the best available database containing information regarding SFTS clusters in China. Accordingly, our findings provide insight into the epidemiological characteristics, risks, and mortality factors of SFTS clusters in China; moreover, they could inform improved strategies and related technical guidelines for the prevention and control of SFTS in China.

## Conclusions

The SFTS clusters were mainly located in central and eastern China, with peaks during summer and autumn. Further, the SFTS clusters showed a high mortality rate and resulted in a high SAR. Most of the index patients had a history of confirmed or suspected tick bite. Their exposed ways are through the routine laboring related with agriculture, such as hunting, cutting wood, seeking medical herbs, picking tea leaves in hills, farming in the fields, seeding, and raising livestock in their yards and surrounding. Contacting the patients’ blood and other fluids can cause secondary transmission, even nosocomial infections. Compared with contacting living patients’ blood, contact with a bleeding corpse was associated with a higher infection risk, which easily contributed to rural community transmission during burial preparation at home. And therefore, technical guidelines and strict policies regarding infection control, case management and corpse decontamination for patients with SFTS should be established and implemented to mitigate transmission and mortality. In addition, delayed diagnosis is a risk factor for SFTS mortality. It is important to increase the rural residents’ awareness of preventing and handling tick bites in endemic areas, as well as enhance diagnostic capacity of the health facilities at the grass-root level, aimed to promote early detection and therefore reduce transmission and mortality caused by SFTSV.

## Data Availability

All data generated or analyzed during this study are included in this published article [and its supplementary information files].

## Abbreviations

NNIDSS: National Notifiable Infectious Diseases Surveillance System
CDPCIS: China Disease Control and Prevention Information System
CFR: Case fatality rate
*CI*: Confidence interval
Ig: Immunoglobulin
*OR*: Odds ratio
PHEE: Public Health Emergency Event
PHEESS: Public Health Emergency Event Surveillance System
PPE: Personal protection equipment
*RR*: Relative risk
SAR: Secondary attack rate
SFTS: Severe fever with thrombocytopenia syndrome
SFTSV: Severe fever with thrombocytopenia syndrome virus
